# Creation of the Person-Centered Wellness Home in Older Adults

**DOI:** 10.1093/geroni/igz055

**Published:** 2020-01-22

**Authors:** Thelma J Mielenz, Melissa Tracy, Haomiao Jia, Laura L Durbin, John P Allegrante, Guedy Arniella, Julie A Sorensen

**Affiliations:** 1 Department of Epidemiology, Mailman School of Public Health, Columbia University, New York, New York; 2 Department of Epidemiology, School of Public Health, University of Albany, Rensselaer, New York; 3 Department of Biostatistics, School of Nursing, Columbia University, New York, New York; 4 Department of Health and Behavior Studies, Teachers College, Columbia University, New York, New York; 5 Community Health and Outreach, Family Health Center of Harlem, New York, New York; 6 Northeast Center for Occupational Health and Safety, Bassett Healthcare Network, Cooperstown, New York

**Keywords:** Chronic illness, Person-centered care, Qualitative analysis, Quantitative research methods, Self-care, Successful aging, Well-being

## Abstract

**Background and Objectives:**

Extending the Patient-Centered Medical Home (PCMH) model into the community may address the poor linkage between medical clinics and underserved communities. Our first of three objectives was to determine if peer leaders and wellness coaches can be the relationship center of wellness care. We evaluated the Self-management Resource Center Small Group Programs (SMRCSGP), plus wellness coaching, as a booster intervention in older adults with chronic diseases. Second, we evaluated the role of personal health records (PHR) prototype as the linkage between the clinic and community. Using input from these two objectives, we lay the groundwork for the Person-centered Wellness Home (PCWH).

**Research Design and Methods:**

Participants enrolled from five South Bronx New York City Housing Authority communities. We conducted a pragmatic, randomized controlled trial using two arms (*n* = 121): (1) SMRCSGP and (2) SMRCSGP plus wellness coaching initiated as a booster after SMRCSGP completion. Adjusted individual growth models compared the slope differences for outcomes. We conducted a social networking analysis on the ties between wellness coaches and participants. PCMH-certified physicians completed in-depth interviews on the PHR prototype. An adaptation from the consensus-workshop model summarized the priority PCWH items.

**Results:**

There was an improvement in self-reported physical functioning (2.0 T-score units higher, *p* = .03) by the wellness coaching group, but the groups did not differ on physical activity. From the social networking analysis, connections were stable over time with wellness-coaches and participants. The Consensus Conference identified eight major components of the PCWH.

**Discussion and Implications:**

Wellness coaching post-SMRCSGP was a booster to physical function, an upstream outcome for physical activity. During the Consensus-Conference, community-based prevention marketing and personal navigators for connecting to a PCMH emerged as novel components. This supports future work in training community health workers as peer leaders to provide evidence-based programs and other PCWH components.

**Translational Significance:**

Eight major components may be important in a person-centered wellness home framework, including: (1) Community-based prevention marketing (CBPM), (2) Personal navigators to obtain insurance or connect to a PCMH, (3) Catalog of evidence-based programs (EBP) in the community, (4) Community health workers (CHW) to be the relationship center of PCWH, (5) Assessment of knowledge done by CHWs included in initial patient assessment, (6) Build self-efficacy by a primer EBP taught by CHW and change culture of health, (7) Ongoing EBP by CHW, and (8) Personal health record dashboard. Further pilot demonstration projects are needed to test these promising components.

Less than 1% of all funds attributed to health care in the United States are spent on prevention efforts to improve overall health and wellness (1). In recent years, the concept of a patient-centered medical home (PCMH) has become increasingly popular and has been implemented in many healthcare settings. Although this treatment approach is a promising strategy for tailoring healthcare to the individual and providing patient support for productively managing their health, the hospital-based PCMH model, fails to successfully link medical practitioners with patient communities or to provide needed support in the community setting. We believe extending the PCMH model of care into the community will encourage person-centered wellness care and the poor linkage between the medical clinics and communities.

Based on the shortcomings and strengths of the chronic care model and the PCMH, we outlined a preliminary framework called the Person-Centered Wellness Home (PCWH) in a commentary/white paper published several years ago (2). Building on two ecological frameworks (general ecological model and the bioecological systems theory), we emphasize community clinic linkages and building community systems in our new PCWH framework (2–5). Person-centered wellness care is a promising population health prevention effort that includes tailored goal-setting for patients, using the patient’s own community resources and preferences, and frequent reassessment of the patient’s needs and goals (2). Self-support wellness care is most efficient when provided in a community setting (2). More specifically, this preliminary PCWH framework included three starting pillars: (1) improvement of chronic disease self-management by utilizing lay leaders and wellness coaches to implement chronic disease self-management programs, (2) measuring how lay leaders and wellness coaches serve as the relationship-center of a wellness home (vs a medical home), and (3) improving clinic-community linkages and promoting self-management by a personal health record (PHR).

Self-management Resource Center Small Group Programs (SMRCSGP) (including programs on general chronic disease and specific conditions: arthritis, diabetes, HIV, chronic pain, and cancer) are structured wellness interventions that encourage self-management in older adults living with chronic conditions and are implemented by lay leaders (6). The Chronic Disease Self-Management Program (CDSMP) is successfully implemented nationally and is considered best practice for chronic disease tertiary prevention (7). Research has indicated that SMRCSGP classes can alleviate some of the burdens associated with chronic diseases by improving health status, health behaviors, and self-efficacy, and by reducing hospitalizations and health care costs (8). It is estimated that $3.3 billion could be saved if the CDSMP reached only 5% of the United States’ adult population (9). Despite the many benefits of SMRCSGP programs, many short-term impacts fade after 9–12 months, including improvements in health care utilization, communication with one’s physician, energy, fatigue, and self-rated health (10). There have been efforts to sustain positive SMRCSGP program benefits with various types of reinforcement, including bi-monthly newsletters, reinforcement courses, automated telephone messages, and listserv e-mail peer-support, but these efforts have not significantly affected program outcomes (11–13). In order to address the fading of SMRCSGP benefits over time, we evaluated **SMRCSGP plus wellness coaching as a booster intervention** in older adults with two or more chronic diseases.

We also conducted a **social network analysis** evaluating the ties between wellness coaches and participants in order to explore the mechanisms through which the wellness coaching intervention might be effective at giving agency to older adults by building relationship-centered wellness care. We constructed a simple model of motivation to comply with behavior change at the individual level using a Social Cognitive Theory (SCT) approach and Network Theory (14,15). From SCT, we used two key concepts: self-efficacy and observational learning. The construct of self-efficacy is well established as affecting health behaviors in general and exercise in particular (16,17). Self-efficacy refers to an individual’s self-confidence that he/she can successfully succeed in an activity. Observational learning occurs when an individual observes others’ behaviors (to include attitudes and beliefs). Although the self-efficacy of others is not observable directly, it can be inferred via others’ actions (physical activity) and social interactions (talking about self-efficacy or exercise). We were interested in the frequency of interactions in the social networks among wellness intervention participants to explore whether increased ties led to higher self-efficacy to exercise. We also examined potential contagion effects to assess whether self-efficacy of others influenced one’s own self-efficacy to exercise.

The final pillar of our preliminary PCWH framework was to explore **the utility of PHR**, which record and track patient health information. In theory, patients could use this information to set personal goals, and could share this with peer leaders, wellness coaches, or community health workers (CHWs) trained in these roles, as well as their primary care physician. This information could be shared with the PCWH team to ensure that all team members are working collectively and productively towards the same goals. However, the utility of a PHR largely depends on the degree to which all PCWH team members find the information helpful, user-friendly and easy to comprehend; thus, we conducted an evaluation of a PHR prototype. Our goal was to conduct a pilot demonstration project testing these three pillars of our preliminary PCWH framework with the a priori assumptions that other key components of the PCWH framework would emerge.

## Method

### Participants, Design, and Procedures

All participants in the study had either completed a 6-week SMRCSGP course within the preceding 2 years on their own, or completed a 6-week SMRCSGP course led by two trained peer leader coaches that we offered prior to the intervention beginning. Written informed consent was obtained from all participants. In addition to completion of an SMRCSGP course, inclusion criteria for participation was as follows: (1) >55 years of age, (2) resided in one of the five identified New York City Housing Authority (NYCHA) public housing developments (Betances, Mill Brook, Mitchel, Mott Haven, and Patterson) or live in the surrounding community, (3) self-report of two or more chronic diseases, (4) cognitively competent, (5) ambulatory (independently or walker/canes), and (6) English- or Spanish-speaking. All necessary study documents were developed, translated, and back-translated into Spanish (e.g., consent forms, surveys). Bilingual research assistants were trained on the recruitment and assessment protocols.

The study comprised a pragmatic, randomized control trial (RCT) with complete block design, using two intervention arms: (1) SMRCSGP and (2) SMRCSGP plus wellness coaching initiated as a booster after SMRCSGP completion. The wellness coaching employed was the Wellcoaches program created by the Wellcoaches Corporation (18). This program draws from many domains including positive psychology, behavioral psychology, the transtheoretical model, motivational interviewing, appreciative inquiry, social psychology, relational cultural theory, adult development, and social cognitive theory/self-efficacy (18). Supporting client autonomy is paramount in the Wellcoaches model (18).

Certified Wellcoaches coaches led three groups (two Spanish and one English) for 24, 1-hr group telephone sessions over 6 months. Classes were convened weekly, except those postponed due to holidays. The wellness self-coaching program asked participants to create a “Wellness Vision,” wherein the participants set monthly and weekly behavioral goals that were agreed upon by participant and coach. Class lesson titles were as follows: taming frenzy, self-compassion, focus, mindfulness, strengths (two-part), motivation, legacy, creativity (two-part), body intelligence (two-part), relationships (two-part), positivity (two-part), meaning (two-part), curiosity (two-part), standard setter (two-part), self-leadership, and your plan to thrive.

**Figure 1.**
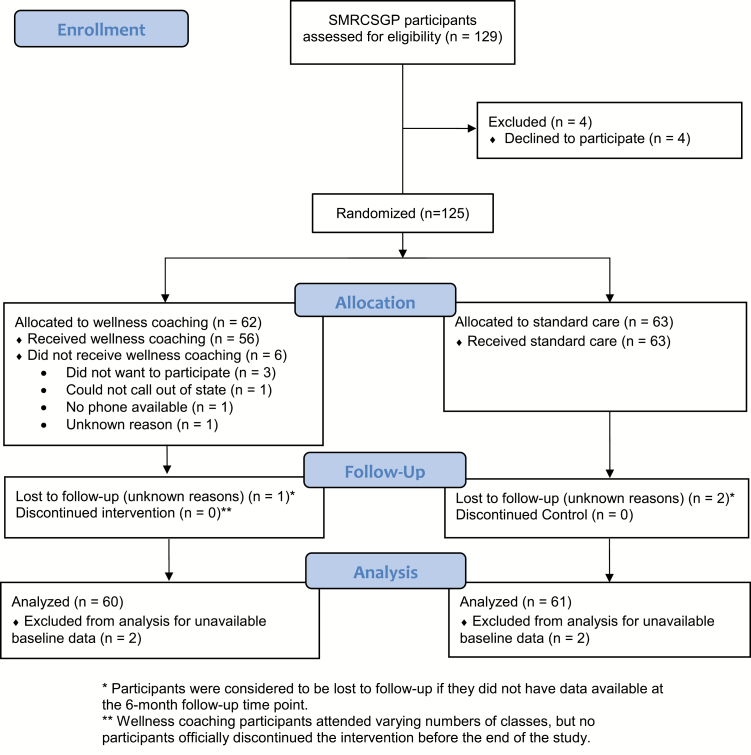
CONSORT flow diagram for wellness coaching SMRCSGP Booster RCT. *Participants were considered to be lost to follow up if they did not have data available at the 6-month follow-up time point.**Wellness coaching participants attended varying numbers of classes, but no participants officially discontinued the intervention before the end of the study.

Consolidated Standards of Reporting Trials (CONSORT) 2010 guidelines were followed during this RCT. Our original sample was 129 participants. After initial dropout, 125 participants were randomized to the wellness coaching intervention or the control group (Figure 1). Control participants only completed an SMRCSGP course and the study surveys. Data were collected monthly and others collected only at baseline, 3-month, and 6-month time points. Data collection was completed in-person with a researcher blinded to study allocation and was collected on paper surveys and later entered into the appropriate databases. Each participant was compensated with $210. This study was approved by the Columbia University Institutional Review Board.

### Measures

#### Personal characteristics

Participant sociodemographic variables recorded at baseline included age in years, sex, education in years, race/ethnicity, living status, and three or more comorbidities.

#### Primary outcomes

Two physical activity measures were the primary outcomes of interest. The Community Health Activities Model Program for Seniors (CHAMPS) was one instrument used to collect information on physical activity. CHAMPS data were collected at baseline, 3 months, and 6 months. CHAMPS is a self-report survey instrument that was developed specifically for older adults and covers 41 activities of varying physical demands (19). The CHAMPS instrument has strong psychometric properties, including validity, test–retest reliability, and sensitivity to change, and has been demonstrated to be appropriate for use in culturally diverse populations (20). CHAMPS includes frequency and hours per week spent engaging in light, moderate, or vigorous exercise-related activities (19).

The other physical activity instrument utilized was the Behavioral Risk Factor Surveillance System (BRFSS) 2011 Physical Activity Rotating Core (PARC), which assesses participants according to the 2008 Physical Activity Guidelines for Americans (21,22). BRFSS physical activity questions were asked monthly starting at baseline and ending at the 6 months follow-up time point. These questions were used to determine if the participants met aerobic physical activity guidelines and if they met both aerobic and muscle strengthening guidelines (23).

#### Secondary outcomes

We had a total of 14 secondary outcomes. Several health domains were measured at baseline, 3 months, and 6 months using the Patient-Reported Outcomes Measurement Information System (PROMIS) v1.0 short form (SF) measures. These included: Emotional Distress-Depression—SF 4a, Fatigue—SF 4a, Pain Behavior—SF 7a, Pain Intensity—SF 3a, Pain Interference—SF 4a, Physical Function—SF 20a), and Sleep Disturbance—SF 4a. PROMIS measures have strong psychometric properties and were specifically developed to be valid and reliable patient-reported measures (24). A higher PROMIS T-score represents more of the concept being measured, with an average score of 50. Other secondary variables that were collected at baseline, 3 months, and 6 months waist circumference (measured in inches by a member of the research team) and medical care questions. These health care utilization measures were taken from the SMRCSGP website.

Certain secondary variables were collected monthly (baseline to 6 months), including falls and self-efficacy for exercise. Falls during the past month were reported on a monthly falls calendar with participants reporting the number of falls and the date a fall occurred (25). Self-efficacy for exercise was assessed on the Resnick Self-Efficacy for Exercise (SEE) Scale, which consists of nine items that ask participants about their confidence to be physically active under a variety of conditions (e.g., the weather is bothering you) on a 10-point confidence scale, with higher scores indicating greater confidence (26,27).

#### Social networking

To assess social networks, each participant was provided with a roster of other individuals in the control and intervention groups (i.e., their specific class ranging from 5 to 22) and asked two questions from Valente’s Network Survey (28). Frequency of interaction was assessed with the question: “On a scale of 1 to 5, where 1 is “not at all,” 3 is “sometimes,” and 5 is “very often,” how would you rate how much you talked to this person in the past week?” Closeness and trust were assessed with the question: “On a scale of 1 to 5, where 1 is “none,” 3 is “somewhat,” and 5 is “a lot,” how would you rate how close you felt to this person over the past week?” A trained research assistant administered the social networking survey once every 4 weeks for 6 months. Since the social network data was most complete at months 4–6 (including ties with coaches), we focused on those three time points when examining the social network structure.

#### The utility of PHR

In order to evaluate the utility of the PHR prototype, researchers conducted interviews with (*n* = 5) primary care physicians registered with the Primary Care Information Project. The Primary Care Information Project is a Health Information Technology Regional Extension Center that facilitates the adoption and use of electronic health record systems amongst primary care physicians in NYC. The Regional Extension Center currently provides IT support and coordination for 4,481 physicians. Of these physicians, 552 (159 practices) have achieved a PCMH certification. Using this list of PCMH-certified physicians and practices, researchers purposively selected five physicians for 90-min in-depth interviews (IDIs) from four different clinics located in the Bronx. Participant physicians were consented. An experienced project coordinator in the primary care practitioner’s clinic conducted interviews using a moderator’s guide developed by the research team. The New York City Department of Health and Mental Hygiene’s Institutional Review Board approved the research.

Questions in the IDI moderator’s guide focused on assessing the utility of the PHR prototype information, the potential benefits of wellness coaching (used in conjunction with the PHR), and the relative appeal of the prototype format. Participants provided feedback on the visual display of information in the prototype dashboard and suggestions for how these infographics might be revised to improve comprehension and rapid relay of health information.

Interviews were transcribed, transcriptions were reviewed for accuracy by the interviewer, and then imported into NVivo, a qualitative analysis software program (29). The research team’s qualitative research consultant then conducted the thematic analysis of interview transcripts using a priori parent and child nodes matched to the qualitative assessment objectives (i.e., utility of the information, potential benefits and relative appeal of the PHR format). Sections of transcript that matched these a priori codes were then highlighted in NVivo and summarized into themes to capture the overarching PCP assessments of the PHR. These themes were reviewed by the PI, interviewer, and qualitative consultant to confirm the thematic conclusions.

#### PCWH consensus workshop model

Exploring workshop models that bridge research and practice in aging, an adaptation from the Cornell Institute for Translational Research on Aging (CITRA) consensus-workshop model was used (30). The Consensus-Workshop was tape recorded and transcribed for the qualitative analysis. Prior to the workshop, research staff reviewed the PCWH literature and summarized data from the Columbia University PCWH pilot study. This information was used to prepare questions and provide information to consensus workshop attendees. The workshop was moderated by J. P. Allegrante, who has previously led consensus model workshops on similar topics. T. J. Mielenz provided an overview of the PCWH model and several consensus panel members (G. Arniella, A. B. Mata, L. L. Durbin, and J. A. Sorensen) were asked to share their experiences working on the PCWH pilot or prior experiences working with the target population. The information was then discussed by participants and used to identify essential components of the PCWH.

### Statistical Analyses

All analyses were conducted using SAS version 9.3.

#### SMRCSGP plus wellness coaching as a booster intervention

We first compared baseline demographics (age, gender, etc.) and outcomes (CHAMPS, PROMIS, etc.) between the intervention and the control group. We compared mean scores using *t* tests and proportions using Chi-square tests. To compare the difference for each outcome measure between the intervention group and the control group, we applied a linear mixed model for continuous measures or a generalized linear mixed model for categorical measures. Let yi,t be outcome measures for person i at time t. The mixed model is in the form of:

$$\begin{array}{ll} h\left(E\left(y_{i,t}\right)\right)= & \beta_{0}+\beta_{1}t+\beta_{2}Group\;+\\ \beta_{3}t\times Group+X{{\;}^{\prime }\;}_{i}\beta +u_{i}, \end{array}$$

where *X*_i_ is a vector of covariates and *h*(*)is an appropriate link function: identity for continuous outcomes and logit for binary outcomes. ui~N0,σ2 is the personal level random effect for repeated measures data. The models compare the trends (i.e., slopes) for each outcome between the two groups (*β*_3_) accounting for the baseline value and adjusted for age, sex, years of education, and comorbidities. Using the effect sizes (5.18 vs 0.58 increase in frequency per week in all listed physical activities) and standard deviation from Stewart et al. for our primary outcome (CHAMPS), our sample of 121 achieves more than 80% power for one of the primary outcomes (CHAMPS).

#### Social networking

For the social network analysis, we focused on ties among the wellness coaching intervention participants (and their coaches) (the “wellness network”). The “egos” of interest are the 56 wellness intervention participants. We also considered the networks based on “frequency of talking” and “closeness/trust” separately. Social network metrics calculated at the network level included: (1) density: the proportion of all possible ties that are actually present and (2) average path length: the average geodesic distance in the network, where a geodesic is the shortest path between two actors. Social network metrics calculated at the ego level included degree centrality, or the number of immediate contacts an actor has in the network. More specifically, we examined unweighted connections (including all connections regardless of strength or direction), connections considered somewhat or very close/frequent, and connections considered very close/frequent. The social networking analysis included network visualizations that were created by NetDraw. In order to assess the effects of social network ties on self-efficacy to exercise, we estimated repeated measures models across all time points for the wellness intervention participants, predicting monthly change in self-efficacy score from their frequency of interaction with other participants and from the average self-efficacy of the other participants with whom they reported interaction or closeness.

#### The utility of the PHR

Discussions were tape-recorded and transcribed and transcriptions were uploaded into NVIVO (2) to facilitate the thematic analysis of focus group transcripts. Prior to sorting, the research team’s Qualitative Researcher, J. A. Sorenson, thoroughly reviewed each of the transcripts to identify and address any potential transcription errors. Following the initial read through, key sections were sorted into the thematic categories and subcategories that were selected prior to the thematic analysis. These a priori categories were developed to reflect the key research questions, such as the utility of the PHR prototype content and formatting.

#### PCWH consensus workshop model

The consensus workshop was recorded and transcribed verbatim, and the transcript was reviewed to extract and summarize recommendations for PCWH model revisions and expansion. The transcript was reviewed using the same process outlined in the methods section, for the analysis of PCP feedback on the PHR prototype.

## Results

### Participant Characteristics

A baseline comparison of intervention and control group characteristics is reported in Table 1. Across both groups, the average age was 72–73 years old, and participants were largely female (75.9–82.8%) and Hispanic (83.6–86.4%). Average educational attainment was approximately 9 years of school and a variety of marital categories were represented. Participants were required to report at least two comorbidities to be included in the study, but approximately half of the sample reported having three or more. Randomization was largely successful, with the only significant discrepancy between the groups being living status; more participants in the intervention group reported living alone (71.2%) than in the control group (58.3%).

**Table 1.** Baseline Characteristics of the Intervention and Control Groups (*n* = 121)*

Characteristics	Intervention (*n* = 60)	Control (*n* = 61)
Demographics		
Age in years (*n* = 115)	72.0 ± 0.94	73.1 ± 0.95
Female sex, % (*n* = 116)	75.9	82.8
Education in years (*n* = 117)	9.0 ± 0.45	8.7 ± 0.61
Race (*n* = 120)		
Non-Hispanic white, %	1.7	1.6
Non-Hispanic black, %	11.9	14.8
Hispanic, %	86.4	83.6
Living status (*n* = 119)^†^		
Lives alone, %	71.2	58.3
Lives with one person, %	28.8	31.7
Lives with more than one person, %	0.0	10.0
Three or more comorbidities, % (*n* = 121)	47.3	47.5
Community Healthy Activities Model Program for Seniors physical activity measures		
Frequency per week of all exercise-related activities (*n* = 120)	7.6 ± 0.61	8.0 ± 0.72
Hours per week of all exercise-related activities (*n* = 120)	24.0 ± 0.93	24.6 ± 1.16
Behavioral Risk Factor Surveillance System physical activity measures		
Met aerobic physical activity guidelines, % (*n* = 92)	65.1	59.2
Met aerobic and strengthening guidelines, % (*n* = 78)	19.5	13.5
Patient-Reported Outcomes Measurement Information System measures		
Depression (*n* = 120)	47.9 ± 1.23	48.3 ± 1.09
Fatigue (*n* = 120)	46.7 ± 1.29	46.6 ± 1.03
Pain behavior (*n* = 120)	51.0 ± 1.44	50.0 ± 1.37
Pain intensity (*n* = 120)	46.1 ± 1.54	45.1 ± 1.40
Pain interference (*n* = 120)	53.2 ± 1.35	51.3 ± 1.08
Physical function (*n* = 120)	39.1 ± 1.40	39.8 ± 1.27
Sleep disturbance (*n* = 120)	51.0 ± 1.24	51.0 ± 1.24
Medical care questions (in the past 6 months)		
Times visiting a physician (*n* = 117)	5.2 ± 2.08	3.3 ± 0.27
Times visiting an emergency department (*n* = 120)	0.6 ± 0.19	0.5 ± 0.09
Times hospitalized for one night or longer (*n* = 119)	0.2 ± 0.08	0.4 ± 0.12
Total nights spent in the hospital (*n* = 120)	0.3 ± 0.16	0.5 ± 0.15
Waist circumference, in inches (*n* = 118)	40.9 ± 0.82	42.3 ± 0.80
Resnick self-efficacy for exercise score (*n* = 118)	6.1 ± 0.32	6.1 ± 0.30
Falls in the past month (*n* = 119)	0.1 ± 0.03	0.1 ± 0.04

*Note*: *Values are the mean ± SE unless otherwise indicated. *T* tests and chi-squared tests were used as appropriate for comparisons. ^†^*p* < .05 for the difference between intervention and control groups.

For both groups, an average of 0.1 falls per person was reported for the last month at baseline. Average waist circumference was indicative of high health risk (40.9–42.3 in). The average self-efficacy to exercise score was 6.1 on a 10-point scale, with a higher score indicating greater confidence that an individual could be physically active. A majority of participants (59.2–65.1%) met the aerobic 2008 Physical Activity Guidelines for Americans, but few met both the aerobic and muscle strengthening guidelines (13.5–19.5%).

### SMRCSGP Plus Wellness Coaching as a Booster Intervention

Employing linear mixed models and generalized linear mixed models (logistic or Poisson as indicated) as appropriate for continuous and categorical outcomes, we found that across the 6 months of our study the intervention and control groups did not vary significantly on any primary physical activity outcomes of interest (CHAMPS and BRFSS measures) in models accounting for the baseline value and adjusted for age, sex, education, comorbidities, and baseline scores (Table 2). The intervention and control groups did vary significantly (*p* = .03) over time on one secondary outcome: the PROMIS physical function variable. Although both groups reported improvements on this measure over time (higher scores indicating that participants can do more and feel better), overall improvement was greater for the wellness coaching intervention group (2.6) than for the control (0.6).

**Table 2.** Model-Based Intention-to-treat Estimates of Outcomes by Group at 6 Months (*n* = 121)*

Outcomes at 6 months	Intervention	Control
Community Healthy Activities Model Program for Seniors physical activity measures		
Frequency per week of all exercise-related activities (*n* = 118)	5.6	6.2
Hours per week of all exercise-related activities (*n* = 118)	21.4	21.6
Behavioral Risk Factor Surveillance System physical activity measures		
Met aerobic physical activity guidelines, % (*n* = 91)	66.0	68.2
Met aerobic and muscle strengthening guidelines, % (*n* = 90)	15.2	9.1
Patient-Reported Outcomes Measurement Information System measures		
Depression (*n* = 118)	47.6	47.4
Fatigue (*n* = 118)	44.0	43.0
Pain behavior (*n* = 118)	50.2	47.0
Pain intensity (*n* = 118)	44.5	41.2
Pain interference (*n* = 117)	49.7	47.4
Physical function (*n* = 118)^†^	41.7	40.4
Sleep disturbance (*n* = 118)	48.8	49.7
Medical care questions (in the past 6 months)		
Times visiting a physician (*n* = 118)	8.5	3.9
Times visiting a hospital emergency department (*n* = 118)	0.4	0.4
Times hospitalized for one night or longer (*n* = 118)	0.3	0.2
Total nights spent in the hospital (*n* = 118)	1.3	3.1
Waist circumference, in inches (*n* = 66)	41.2	42.0
Resnick self-efficacy for exercise score (*n* = 122)	7.0	6.7
Falls in the past month (*n* = 117)	0.08	0.05

*Note*: *All models account for the baseline value and adjusted for age, sex, education, and comorbidities. Linear mixed models are employed for continuous outcomes and generalized linear mixed models are employed for categorical outcomes (logistic or Poisson as indicated).

^†^ *p* < .05 for the difference between intervention and control groups over time.

### Social Networking

Network connections between the wellness intervention participants and their wellness coaches remained fairly stable over time, with similar network density, distributions of tie strength, and ego centrality (Table 3). The number of social ties to other participants in the previous month was not associated with change in self-efficacy to exercise scores among the wellness intervention participants, nor was the average self-efficacy scores among social ties, even when restricting to those ties reported as “very close” or who talked “very often” (Table 4).

**Table 3.** Descriptive Statistics for the Wellness Network and for Ego Characteristics of the Wellness Network (months 4–6; *n* = 55 intervention participants)

Frequency of talking	Month 4		Month 5		Month 6	
	*N* or Mean	% or *SD*	*N* or Mean	% or *SD*	*N* or Mean	% or *SD*
**Overall characteristics**						
Total number of ties	863		927		879	
Number of “sometimes” ties^a^	145	16.8	153	16.5	147	16.7
Number of “very often” ties^b^	17	2.0	29	3.1	18	2.1
Network density	0.334		0.335		0.329	
Average path length	1.01		1.49		1.03	
**Ego characteristics**						
Total number of egos	55		55		55	
Degree centrality, unweighted	19.1	5.7	19.4	5.7	19.2	5.7
Number of “sometimes” or “very often” ties^c^	3.7	2.5	4.1	2.8	3.7	2.5
Number of “very often” ties^b^	0.55	0.98	0.80	1.4	0.56	1.0

*Note*: ^a^Frequency of talking in the past week was reported as “sometimes” for these ties.

^b^Frequency of talking in the past week was reported as “very often” for these ties.

^c^Frequency of talking in the past week was reported as “sometimes” or “very often” for these ties.

**Table 4.** Repeated Measures Models Predicting Change in Monthly Self-efficacy to Exercise Scores Across All Time Points (*n* = 56 wellness intervention participants), Based on Frequency of Talking in Previous Month and Average Self-efficacy of Connections in Previous Month

	Beta	*SE*	*p*-value
Frequency of talking			
Degree centrality	−0.009	0.034	.786
Number of “sometimes” or “very often” ties^a^	−0.040	0.058	.492
Number of “very often” ties^b^	0.076	0.107	.479
Average self-efficacy of alters^c^			
Among all ties	0.105	0.548	.848
Among close or frequent ties	0.051	0.099	.602
Among very close or very frequent ties	0.080	0.098	.413

*Note*: ^a^Frequency of talking in the past week was reported as “sometimes” or “very often” for these ties.

^b^Frequency of talking in the past week was reported as “very often” for these ties.

^c^For each wellness intervention participant, change in monthly self-efficacy ratings is predicted from the average self-efficacy ratings of their reported connections in the previous month.

### The Utility of PHR

Physicians provided many valuable suggestions for improving PHR prototype usability, content, and format. The physicians identified several components of the PHR that might be difficult for patients to understand. Physicians also stated that the language should be simplified. Physicians felt symbols indicating whether health metrics are in the normal range were unclear. Physicians perceived that it was important information to include information on the patient’s lifestyle, such as the level and type of physical activity, diet (as well as number of calories consumed) and stress. Additionally, useful clinical information would include weight, BMI, blood pressure, medication compliance, A1C changes over time and the date of their last medical appointment. One physician also suggested that an assessment of the patient’s “stage of change” in relation to lifestyle changes would provide a useful global measure of patient progress.

Physicians also mentioned that patient goals should be provided in the PHR, so that both physicians and nurses could reinforce the importance of these goals. The addition of a section where the patient could list barriers to healthy lifestyle changes was also suggested. These barriers could then be discussed and addressed by the patient, physician, and nurses. Adding contact information for the wellness programs and a short description of wellness class topics in the PHR was another suggested addition. Physicians also identified a number of informational items that they felt were irrelevant and could be removed. One physician indicated that it was not important to know if the patient is smoking, stating “if you know that the patient is smoking, there is no need to go at it more than one time, unless the patient wants to stop smoking.” Key components of the PHR should include: (1) health data in chart format (i.e., visual depictions of trends with anchors that depict “normal” health levels, emojis to demonstrate a warning and information headings for lay readers), (2) behavior change data (e.g., physical activity, level of physical activity confidence, diet, smoking, and alcohol consumption), (3) contact information for the lay health leader and evidence-based wellness programs in the community, (4) a list of patient questions, and (5) a list of patient goals. Other formatting suggestions included limiting the document to one page, reducing duplication, and using a large font size.

Unfortunately, there was very little physician consensus on how often they would like to receive the information depicted in the PHR. Some said weekly, others indicated every few weeks, while two of the physicians preferred to see the information monthly or every few months. However, physicians did overwhelmingly agree that it would be most useful to have the patient bring this information at the time of their visit. Physicians said they preferred this option, as it forces the patient to be more involved and proactive in health maintenance efforts. Several physicians also mentioned the benefit of being able to scan the PHR at the time of the visit so that it could be included with the patient’s electronic medical records. Physicians felt this information should be made available to nurses.

### PCWH Consensus Workshop Model

**Figure 2.**
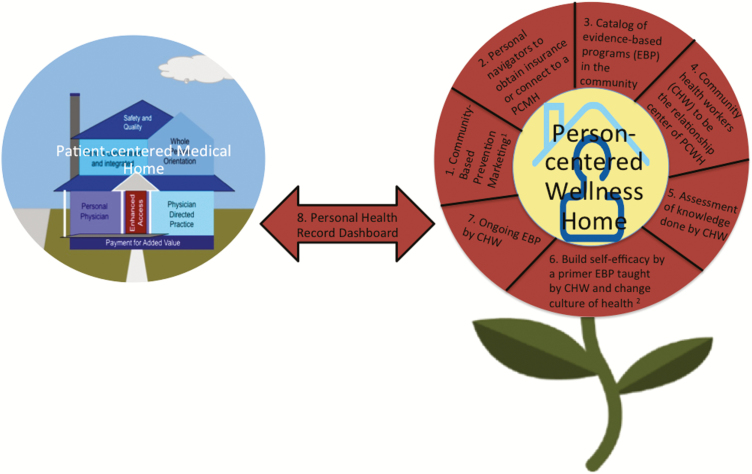
The components of the Person-centered Wellness Home. Adapted from refs. (31,42,43).

After the Consensus-Workshop was convened, we summarized the highest priority PCWH components discussed by the group. These eight components are presented below and in Figure 2. 

1. **Community-based prevention marketing (CBPM).** CBPM uses “a community-driven program planning framework that applies social marketing concepts and techniques to the development of health behavior interventions” (31). Using social marketing as a planning framework, participants should be actively involved in tailoring the PCWH model to meet their specific needs. Targeted populations are actively involved in tailoring program objectives and components, as well as providing direction on how to best implement these programs in their communities (31).
2. **Personal navigators to obtain insurance or connect to a PCMH.** PCWH programs should be connected with primary care clinics that are certified medical homes. For individuals who are not connected to a PCMH, their alternate source of medical care, typically emergency departments (EDs), should steer these patients to a local PCMH. In addition to connecting them with a primary care physician working within a certified PCMH program, individuals attending EDs with chronic health conditions should be assisted with applying for and learning how to use healthcare insurance. This assistance could be provided through health insurance navigators, which may be new to these communities, but which could be promoted through the EDs. These health insurance navigators can be CHWs, described further in 4 below, with care coordination and chronic disease management training under Medicare Part B’s chronic care management services (1).
3. **Catalog of evidence-based programs (EBP) in the community.** In addition to the PCMH, which would function as the core connection between the provider and patient, the PCWH would include several evidence-based, chronic disease management programs. These would be placed within patient communities and managed by the community health workers (e.g., health insurance navigators, CDSMP peer leaders or health coaches), who would be trained to provide these health maintenance programs. A list of trainers specializing in various evidence-based programs around the state would be provided to clinicians, who could refer their patients accordingly. Physicians could use the directory to refer patients to disease management programs and community health workers could use the directory to find training or networking opportunities.
4. **CHW to be the relationship center of PCWH.** Efforts are ongoing to integrate CHWs into the PCMH (31). Their services can be reimbursed under the Affordable Care Act (ACA). The CHWs can provide relationship-centered care in the community or the PCWH and serve as the link to the clinic or the PCMH (2).
5. **Assessment of knowledge done by CHWs included in initial patient assessment.** In order to assist the patient in understanding their condition and ability to prevent and productively manage existing health conditions, it was suggested that patients participate in an orientation with their physician and community health coach. This would help them understand their health issues and provide them with opportunities to ask questions. The orientation would also allow the provider and community health coach to assess how well the patient understands their chronic disease issues and how to manage them.
6. **Build self-efficacy by a primer EBP taught by CHW and change culture of health.** Reviewing the CHWs’ roles and related tasks in the New York State Community Health Initiative many of the tasks build self-efficacy (e.g., promoting health literacy, coaching on problem-solving, supportive counseling, action plan implementation and leading support groups) ultimately leading to a change in the culture of health (32,33).
7. **Ongoing EBP by CHW.** CHWs can be trained to be the peer leaders for EBP and provide a menu of EBP on important older adult topics (e.g., chronic disease self-management, pain management, fall prevention, and caregiving).
8. **PHR dashboard.** In addition to the provision of support in the primary care setting and in patient communities, a PHR dashboard could be used to list and track the patient’s chronic disease management goals. The health dashboard would provide key health indicators, as well as important chronic disease management goals tailored to the individual. The information would help the patient track their health goal activities in between clinic appointments and information could facilitate communication between the patient’s healthcare provider and community health trainers or coaches.

The following Barriers to Implementing the PCWH Model also emerged from our Consensus-Workshop.

1. **Lack of resources**. One of the participants stated there is little funding available to train health coaches and to pay them to provide the services, and there is no available space for workshops or classes. Many individuals lack healthcare insurance or access to a primary care physician, providing further challenges for individuals who might benefit the most from a PCWH model.
2. **Sociocultural beliefs.** Participants indicated that patients tend to be fatalistic, believing there is nothing they can do to prevent or control their chronic health issues. A proactive stance regarding healthcare is also heavily influenced by patient communities which, according to community experts, often view having health problems as a “given.”
3. **Sustainability.** This will require the identification and dedication of long-term resources that will ensure trained community health coaches stay with the program, that the infrastructure to support the clinical and community services stays intact and that the patients stay engaged in these programs long enough for investments to result in long-term health improvements.
4. **Addressing other PCWH barriers.** These include lack of financial resources, mental health issues, or familial responsibilities, such as caring for someone with special needs. These personal challenges can markedly limit an individual’s time for self-care. One strategy for addressing these community-specific PCWH barriers would be to ask community members to identify solutions they have developed to address these challenges. If they can share solutions they have developed or have heard others discuss, then it is likely these options will be culturally and environmentally suitable. Locations for chronic disease management classes should also be geared towards public venues such as schools, churches, or community centers that are most appealing and convenient for patients.

## Discussion

Wellness coaching post-SMRCSGP was a booster to physical function, a plausible upstream outcome for physical activity (34). Weekly wellness coaching demonstrated its potential to be a self-efficacy booster, although social network ties between participants and between participants and coaches did not appear to facilitate self-efficacy to exercise. Control and wellness coaching intervention participants did not vary on any of our other health and well-being outcome measures.

During the Consensus-Conference, Community-Based Prevention Marketing and personal navigators to obtain insurance or connect to a PCMH emerged as novel components of the PCWH. A CHW trained in SMRCSGP or equivalent evidence-based program is thus capable of implementing the majority of the remaining PCWH components (3 through 7 in our figure), which can be reimbursed under the Affordable Care Act. Direct linkage to a PCMH is required only for the PHR Dashboard component, illustrating how community health workers can be cost-effective wellness providers in the context of low-income and disadvantaged communities. The Society of Behavioral Medicine made recommendations to integrate CHWs into PCMH by broadening the scope of the services and clearly defining the tasks (e.g., Health insurance navigators, CDSMP peer leaders, or health coaches) that CHWs provide (35). Work is currently underway to identify the barriers and facilitators to utilizing CHWs in PCMH (36).

Physician feedback demonstrated that the concept of a PHR record was sound and could facilitate improved and efficient communication regarding the patient’s health trends, goals and barriers to improvement. Data presentation preferences included depicting trends and separating the sections of the PHR into distinct areas, such as patient information, patient goals, patient questions, and wellness program content and contact information. Uptake of patient portals in PCMH has remained poor (37). PCMH physicians reported that accessing and training for the PHR portals outside of the clinic would increase updated and decrease the burden to the clinic to facilitate this (37). The evaluation of PHR to influence wellness and physician promotion of prevention care is underway and positive (38).

In general, the PCMH physicians indicated the concept of a PCWH would greatly improve patients’ abilities to be engaged and proactive in managing and improving their health. By establishing wellness programs within the community, patients could more easily and frequently take advantage of these services, which would in turn improve their ability to maintain healthy lifestyle changes. These localized community health services would also allow physicians to dedicate precious patient interaction time to other issues that need to be addressed.

Feedback from researchers and community experts at the PCWH Consensus workshop indicates that the PCWH should incorporate additional steps that connect chronic disease management patients with a PCMH and healthcare insurance. The addition of a patient orientation to the PCWH program would allow providers to establish a productive baseline with their patients so they can better understand their health conditions and how proactive health behaviors can benefit them. Lastly, community members and patients should be actively involved in the development and implementation of local PCWH programs. By participating in the process of PCWH program development, these programs are more likely to both appeal to patients and be sustainable.

This study had several limitations. The high proportion of Hispanic and female participants, low levels of educational attainment and increased walking done in New York City’s older adults may limit generalizability of our results to other populations (39). Second, due to time constraints, our 24-session wellness coaching intervention had to be condensed from 1 year to 6 months, with classes provided every week rather than twice a month. If the sessions had been spaced according to the original plan of the Wellcoaches Corporation, then there would have been more time for the participants to put what they learned into practice. Wellness coaches are currently frequently used in level 3 PCMH (40). Finally, characteristics of social ties other than frequency of interaction and closeness/trust may be relevant for behavior change (e.g., relationship type) but were not assessed in this study (41). Despite these limitations, our study was strengthened by the use of primary and secondary outcome measures with strong psychometric properties. Moreover, our dropout and loss-to-follow-up rates for an intervention study with a duration of 6 months are also strong, with only four participants unable to be included in the analysis.

## Conclusion

Future steps for further developing the PCWH model and building on the suggestions made in the consensus workshop include organizing a consensus model conference that would include a wider spectrum of participants including participant stakeholders. This conference would develop an algorithm for implementing PCWH programs in at-risk communities. Another demonstration pilot focusing on a majority of the specific identified components of the PCWH model is being planned, using current falls programming lay leaders implementing falls screening, a fall risk prevention self-efficacy focused program followed by a fall risk prevention exercise focused program.
