# Creation of the Person-Centered Wellness Home in Older Adults

**DOI:** 10.1093/geroni/igaa005

**Published:** 2020-03-18

**Authors:** Thelma J Mielenz, Melissa Tracy, Haomiao Jia, Laura L Durbin, John P Allegrante, Guedy Arniella, Julie A Sorenson

**Affiliations:** 1 Department of Epidemiology, Mailman School of Public Health, Columbia University, New York, New York; 2 Department of Epidemiology, School of Public Health, University of Albany, Rensselaer, New York; 3 Department of Biostatistics, School of Nursing, Columbia University, New York, New York; 4 Department of Health and Behavior Studies, Teachers College, Columbia University, New York, New York; 5 Community Health and Outreach, Family Health Center of Harlem, New York, New York; 6 Northeast Center for Occupational Health and Safety, Bassett Healthcare Network, Cooperstown, New York

The article “Creation of the Person-Centered Wellness Home in Older Adults” (*Innovation in Aging*, igz055, https://doi.org/10.1093/geroni/igz055) was published in the incorrect issue and should have been published in Volume 4 Issue 1. This has now been updated.

